# Circadian alignment of food intake and glycaemic control by time-restricted eating: A systematic review and meta-analysis

**DOI:** 10.1007/s11154-023-09853-x

**Published:** 2023-11-22

**Authors:** Susana Rovira-Llopis, Clara Luna-Marco, Laura Perea-Galera, Celia Bañuls, Carlos Morillas, Victor M. Victor

**Affiliations:** 1 Departamento de Fisiologia, Facultad de Medicina y Odontologia, Universidad de Valencia - INCLIVA Biomedical Research Institute, Valencia, Spain; 2 Service of Endocrinology and Nutrition, University Hospital Doctor Peset, Foundation for the Promotion of Health and Biomedical Research in the Valencian Region (FISABIO), Valencia, Spain; 3 CIBERehd - Department of Pharmacology, University of Valencia, Valencia, Spain

**Keywords:** Time-restricted eating, Intermittent fasting, Glucose, Insulin, Diabetes, HbA1c

## Abstract

Daily rhythms of metabolic function are supported by molecular circadian clock systems that are strongly regulated by feeding and fasting. Intermittent fasting diets have been associated with weight loss and improved metabolism. However, the effects of time-restricted eating (TRE) on glycemic parameters are still under debate. In this review, we aim to systematically analyze the effects of TRE on glycemic parameters. We searched on PubMed, EMBASE, and the Cochrane Library for controlled studies in which subjects followed TRE for at least 4 weeks. 20 studies were included in the qualitative systematic review, and 18 studies (n = 1169 subjects) were included in the meta-analysis. Overall, TRE had no significant effect on fasting glucose (Hedges’s g = -0.08; 95% CI:-0.31,0.16; p = 0.52), but it did reduce HbA1c levels (Hedges’s g = -0.27; 95% CI: -0.47, -0.06; p = 0.01). TRE significantly reduced fasting insulin (Hedges’s g = -0.40; 95% CI: -0.73,-0.08; p = 0.01) and showed a tendency to decrease HOMA-IR (Hedges’s g = -0.32; 95% CI:-0.66,0.02; p = 0.06). Interestingly, a cumulative analysis showed that the beneficial effects of TRE regarding glucose levels were less apparent as studies with later TRE windows (lTRE) were being included. Indeed, a subgroup analysis of the early TRE (eTRE) studies revealed that fasting glucose was significantly reduced by eTRE (Hedges’s g = -0.38; 95% CI:-0.62, -0.14; p < 0.01). Our meta-analysis suggests that TRE can reduce HbA1c and insulin levels, and that timing of food intake is a crucial factor in the metabolic benefit of TRE, as only eTRE is capable of reducing fasting glucose levels in subjects with overweight or obesity.

PROSPERO registration number CRD42023405946.

**Supplementary Information:**

The online version contains supplementary material available at 10.1007/s11154-023-09853-x.

## Introduction

Modern society is characterized in general by the ad libitum availability of food, continuous exposure to artificial light sources (especially devices with a screen), and continuous disruption of sleep and daily activities. All these factors disturb the day-night biological rhythm and are associated with the development of cardiometabolic diseases, especially type 2 diabetes [1, 2]. Intermittent fasting (IF) is a dietary regimen that consists of alternating periods of fasting and eating, either within a week – e.g. alternate-day fasting – or within a day – e.g. time-restricted eating (TRE) –. In recent years IF has become popular and many studies have been conducted to assess its effectiveness in improving the metabolic alterations induced by obesity and metabolic disorders.

TRE involves ad libitum eating in controlled time windows, emphasizing meal timing over calorie intake. A typical TRE protocol consists of 16:8 time windows [3], with the daytime feeding cycle comprising the shorter period (8 h), and a prolonged fasting period spanning the evening and continuing overnight (16 h). However, other meal timing windows have been tested in interventional studies [4, 5], and common fasting practices for religious purposes, such as the practice of Ramadan, involve an overnight eating window [6]. Some beneficial results of TRE have been described at the metabolic level in people with obesity or overweight, including an increase in lipid oxidation [7], a decrease in plasma glucose levels [8], and an improvement of insulin sensitivity [9]. Although the results obtained to date have been promising, some of the studies in question applied very short meal intervals (4 h, 6 h) [4, 5], which hinders their application to daily life. There are limited studies in subjects with type 2 diabetes [10, 11], and so the effects of TRE on metabolic health in that specific population remain unclear. In this sense, Andriessen et al. showed that three weeks of TRE improved mean 24 h glucose levels and time in range in adults with type 2 diabetes, but it did not improve insulin sensitivity [12].

TRE has beneficial effects at the metabolic level, both when there is weight loss [13] and when there is not [9], suggesting that the metabolic benefit may be due to other mechanisms underlying TRE and not solely to body weight changes. In this sense, some studies have pointed out that, compared to control lean subjects, people who have metabolic problems also develop alterations at the circadian cycle level in terms of the rhythmicity of metabolic processes, such as mitochondrial oxidative capacity [14], glucose homeostasis [15] and oxidation of substrates [14, 16]. It has, therefore, been proposed that disruption of the circadian rhythms can contribute to an impaired balance of substrate utilization and availability, which in turn is associated with metabolic diseases [17]. It has also been suggested that alterations in metabolic rhythmicity are the result of an altered cycle of feeding and fasting. Therefore, restricting food intake during the day to extend the fasting period may improve glycemic control.

Over the past few years, systematic review and meta-analyses have been conducted in order to shed light on the potential of TRE regimens to improve glucose metabolism related endpoints in different populations [6, 18, 19]. Although important contributions have been made in the field, it is necessary an update, since in the last 4 years a growing number of interventional studies in humans have been conducted. As a reference, only in 2022, 10 randomized clinical trials finalized and published results [11, 20–28]. Therefore, this systematic review was undertaken to evaluate whether TRE has beneficial effects on glycemic parameters. Further understanding of this subject would undoubtedly aid the development of nutrition strategies that prevent and even halt the development of metabolic diseases such as type 2 diabetes.

## Methods

This systematic review was performed according to the guidelines of The Preferred Reporting Items for Systematic Reviews and Meta-Analyses (PRISMA)[29]. The study has been registered in the International prospective register of systematic reviews (PROSPERO) with the registration number CRD42023405946.

### Search strategy

We selected relevant studies published from inception to 1st January 2023 in 3 different databases: PubMed, EMBASE, and the Cochrane Library.

Several search terms were combined, including "time-restricted eating", "time-restricted feeding", "prolonged nightly fasting" or "prolonged overnight fasting" and "glycaemic control", "HbA1c", "glucose" or "diabetes". The details of the search terms used in each database are included in the Supplementary file.

Search and data extraction was independently carried out by two researchers (SRL, CLM). Discrepancies were resolved by consensus discussion with a third investigator (LPG).

### Inclusion/exclusion criteria

Studies that met the following criteria were included: controlled studies in which the control group followed a dietary regimen without temporal restriction, studies with a parallel-arm or with a crossover design, in adults (≥ 18 years of age) assigned an intervention consisting of daily TRE, including a fasting window of between 14 and 18 h/day, associated or not with energy restriction and with a duration of 4 to 14 weeks. Both randomized and non-randomized designs were included. Studies were required to have assessed fasting glucose or HbA1c. There were no restrictions based on sex, race or body mass index (BMI).

We excluded studies with other IF protocols, such as Ramadan or 5:2 diets, and where other interventions were applied in addition to the TRE, as well as those involving co-administration of drugs or studies lacking a control group.

### Outcomes

The primary outcome of this systematic review and meta-analysis was to determine the effects of TRE treatment on glucose metabolism by analyzing pre- and -post differences in fasting glucose and HbA1c.

Secondary outcomes were changes in fasting insulin and the Homeostasis Model Assessment—Insulin Resistance (HOMA-IR) index.

### Data collection

We extracted the following information from each study: first author name and year of publication, study design, study population, duration of the intervention, fasting:feeding hours, type of diet, eating window, control group characteristics, number of subjects enrolled in each arm, age, gender and main outcome/s. For the meta-analysis, pre- and post- mean and standard deviation (SD) of fasting glucose, HbA1c, fasting insulin and HOMA-IR were collected. If post mean and SD data were not reported, we recorded the difference in means and the SD of the difference. If neither of these data was available, it was requested from the authors.

### Data synthesis

Comprehensive Meta-Analysis software version 4 [30] was used for all analyses. Due to inevitable heterogeneity between studies, the random-effects model was chosen for all analyses. To assess the heterogeneity between and within the selected studies, I^2^ and Tau (  ) statistics were used, respectively.

When needed, 95% CI was converted to SD according to the calculations outlined in the Cochrane Handbook for Systematic Reviews of Interventions [31].

We estimated the effect size using Hedges’s g that estimates the difference in means and uses pooled weighted standard deviations to obtain the corrected effect size. This measure is sensitive enough to detect differences in studies with small sample size (e.g. n lower than 20). Further details of Hedges’s g can be obtained from the original publication [32].

### Quality assessment

For randomized controlled trials (RCTs), we used the risk of bias according to the Cochrane risk assessment tool (RoB-2) [33]. For non-RCTs, we used the ROBINS-I tool [34]. SRL and CB independently assessed the quality of the studies and agreed with the results.

## Results

### Selected studies

The initial database search identified a total of 848 articles. After removing duplicates, 504 records were screened for eligibility. Following the removal of reviews, other types of studies and those performed in animal models or in vitro, a total of 100 articles were selected for full-text evaluation.

**Fig. 1:**
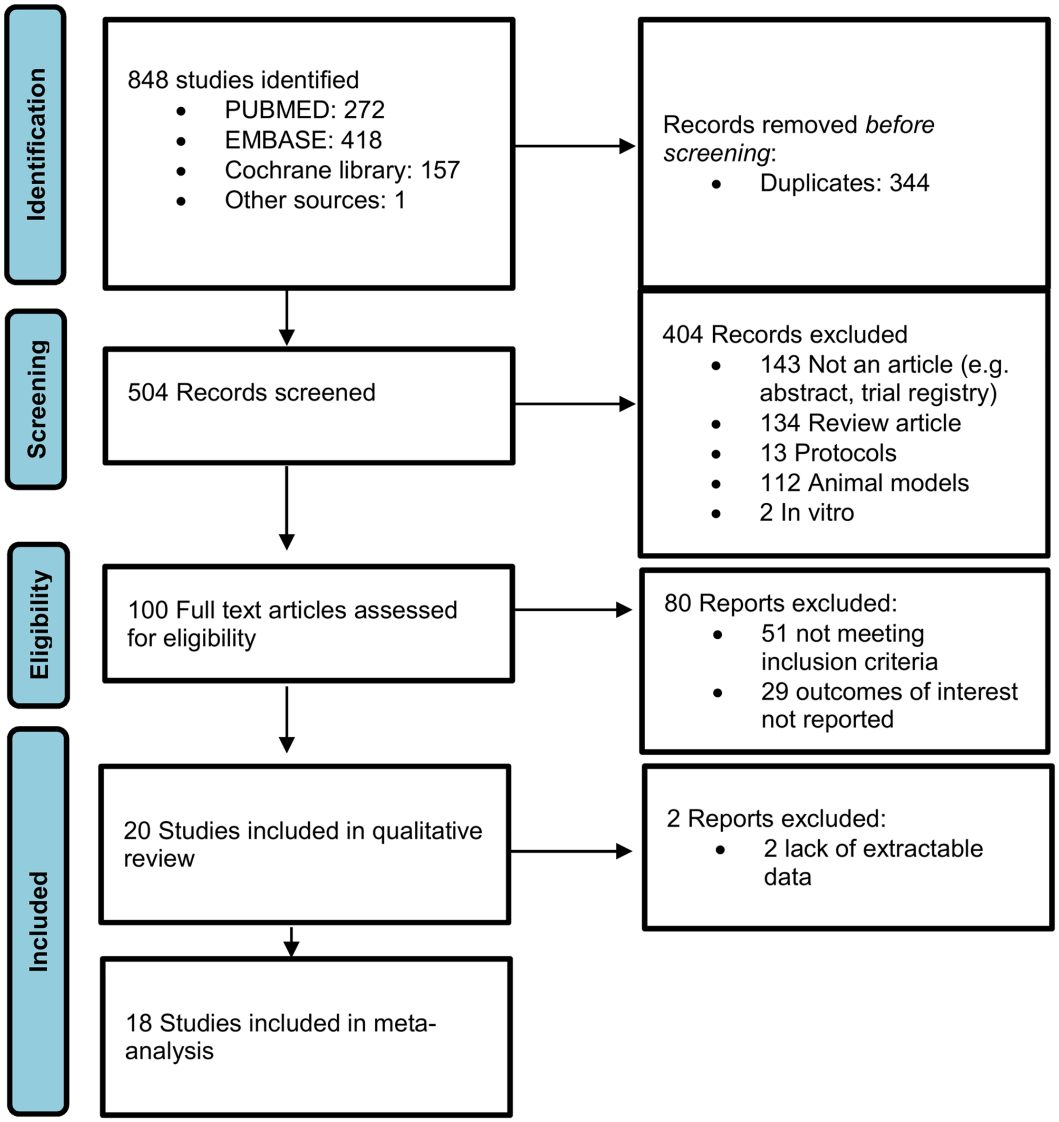
PRISMA flow diagram describing the process of study selection

Of these 100 articles, 51 were excluded because they did not meet the inclusion criteria, and 29 were ruled out because the outcomes of interest were not reported, leaving a final 20 articles to be included in the systematic review. Figure 1 displays the flow diagram of the selection of the studies. Further details of the reason for excluding each study can be found in Supplementary Table 1.

### Quality assessment

**Fig. 2:**
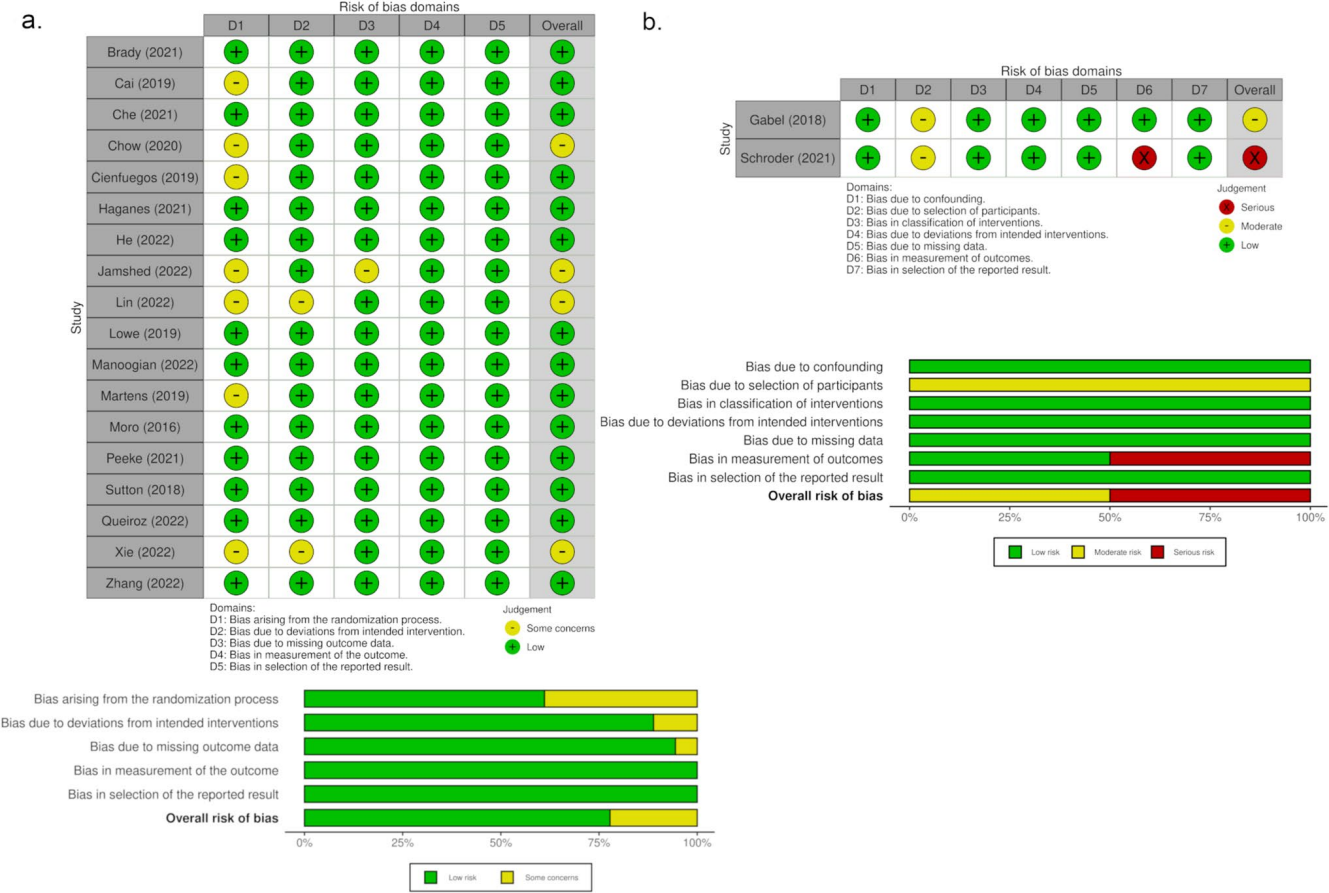
Quality assessment. **a**. Estimated risk of bias for RCTs using the RoB 2 tool. **b**. Estimated risk of bias for non-RCTs using the ROBINS-I tool

The results for quality appraisal are displayed in Fig. 2A for RCTs and Fig. 2B for non-RCT. Of the 18 RCTs, 14 were assessed as high quality and 4 have some concerns in the overall judgment. They were in part due to bias arising from the randomization process [4, 22, 23, 26], but also due to deviations from the intended interventions [23, 26] and concerns about missing outcome data [22]. For the non-RCTs, we found some concerns in the study by Gabel et al. due to the selection of participants. The control group was historical, meaning that subjects were recruited in a previous period, with a lapse of up to five years [3]. This could have influenced the selection of food due to seasonality, and the subjects’ knowledge about weight control. Serious risk of bias was estimated for the study of Schroder et al. due to the selection of outcomes [35].

### Characteristics of the included studies

Our qualitative analysis included the final 20 selected studies; however, two of the studies were excluded from the meta-analysis due to a lack of extractable data [26, 27]. The main findings of the two studies in question are summarized in Supplementary Table 2.

The 18 studies selected for meta-analysis are summarized in Table 1. They included a total of 1169 subjects, and sixteen studies were RCT [4, 5, 9, 11, 20–25, 36–41], of which two had a cross-over design [9, 39]. The remaining two studies were non-RCT; one recruited a control group in parallel to the TRE group [35], and the other used a historical control group from a previous cohort recruited by the same authors [3].

**Table 1:** Characteristics of the included studies

**Author (year)**	**Study design and groups**	**Population**	**Duration (weeks)**	**TRE hours Fast:Fed**	**Calorie Restriction**	**Eating window**	**Control group**	**n (control/TRE)**	**Age group**	**Sex**	**Main outcome**
Brady et al. (2021) [40]	Randomized, C/TRE	Runners	8	16:8	No (ad libitum)	12am-8 pm	Habitual diet	7/10	Middle-aged	M	Change in body mass
Cai et al. (2019) [37]	Randomized, C/TRE	NAFLD patients BMI > 24	12	16:8	No (diet counseling)	Self-selected	20% CR no TRE	79/95	Young	M/F	BW, waist circumference, body composition, liver stiffness, cholesterol, LDL, HDL, TG, glucose
Che et al. (2021) [11]	Randomized, C/TRE	Type 2 diabetes	12	14:10	No	8am-6 pm	Habitual diet	50/54	Middle-aged	M/F	Glucose, HbA1c, BW, HOMA-IR, CVD markers
Chow et al. (2020) [4]	Randomized, C/TRE	Overweight or obesity	12	16:8	No	Self-selected (10:30am-6:30 pm)	> 15 h fed	9/11	Middle-aged	M/F	BW, body composition, lipids, blood pressure, 2-h oral glucose tolerance, 2-week continuous glucose monitoring, and 2-week physical activity
Cienfuegos et al. (2020) [5]	Randomized, C/4 h-TRE/6 h-TRE	Obesity BMI 30–49.9	8	20:4 or 18:6	No (ad libitum)	1 pm-7 pm	Habitual diet	14/19(6 h)	Middle-aged	M/F	BW
Gabel et al. (2018) [3]	Not Randomized, C/TRE	Obesity	12	16:8	No	10am-6 pm	Historical	23/23	Middle-aged	M/F	BW
Haganes et al. (2022) [20]	Randomized, 4 groups: C/TRE/HIIT/TRE + HIIT	Overweight/Obesity BMI ≥ 27	7	14:10	No	Self-selected (before 8 pm)	Habitual diet	33/33	Middle-aged	F	Total area under the curve in glucose tolerance test
He et al. (2022) [21]	Randomized4 groups: C (LCD)/eTRE/lTRE/LCD + TRE	Metabolic syndrome	12	16:8	- TRE No, Ad libitum - Control Yes	8am-4 pm (earlyTRE) 12 pm-8 pm (lateTRE)	LCD	55/e-38/l-17	Middle-aged	M/F	BW and abdominal fat area
Jamshed et al. (2022) [22]	Randomized, C/TRE	Obesity BMI ≥ 30	14	16:8	Yes	7am-3 pm	12:12 CR (fast:fed)	45/45	Middle-aged	M/F	weight loss and fat loss
Lin et al. (2022) [23]	Randomized, C/TRE	Overweight BMI ≥ 24	8	16:8	Yes	10am-6 pm or 12 pm-8 pm	Traditional weight loss method w/o TRE	33/30	Middle-aged	F	BW, body composition, biochemical parameters
Lowe et al. (2020) [38]	Randomized, C/TRE	Overweight/ObesityBMI 27 to 43	12	16:8	No (*ab libitum*)	12 pm-8 pm	Consistent meal timing, no TRE	57/59	Middle-aged	M/F	BW
Manoogian et al. (2022) [24]	Randomized, C/TRE	Volunteers from Fire-Rescue Department, 71% had at least one cardiometabolic risk factor	12	14:10	No, but Mediterranean diet and counseling	Self-selected 9am-3 pm	Standard care (Mediterranean diet)	67/70	Middle-aged	M/F	Feasibility and glucose homeostasis
Martens et al. (2019) [39]	Randomized crossover, C/TRE	Healthy normal weight	6 cross 6	16:8	No	Self-selected (10:30am- 6:30 pm)	Habitual diet	22/22 (same subjects)	Older adults	M/F	Feasibility, endothelial function
Moro et al. (2016) [36]	Randomized, C/TRE	Resistance-trained males	8	16:8	No	1 pm-9 pm	12:12 (fast:fed)	17/17	Young	M	Fat mass and fat-free mass
Peeke et al. (2021) [41]	Randomized, pilot, C/TRE	Obesity BMI ≥ 30	8	14:10	Yes	variable 9am-12 pm and 5 pm-8 pm	12:12 CR (fast:fed)	30/30	Middle-aged	M/F	BW
Schroder et al. (2021) [35]	Non-randomized, C/TRE	Obesity BMI ≥ 30	12	16:8	No (ad libitum)	12am-8 pm	Habitual diet	12/20	Middle-aged	F	BW, body composition
Sutton et al. (2018) [9]	Randomized crossover, C/TRE	Pre-diabetes and overweight/obesity	5 cross 5	18:6	No (meals provided)	9am-3 pm	12:12 (fast:fed)	8/8 (same subjects)	Older adults	M	Glucose tolerance, postprandial insulin, and insulin sensitivity as measured using a 3-h oral glucose tolerance test
Queiroz et al. (2022) [25]	Randomized, C/eTRE/lTRE	Overweight/Obesity BMI = 25–34.9	8	16:8	Yes	8am-4 pm ("early") or 12am-8 pm ("delayed")	12:12 CR (fast:fed)	13/e13/l11	Young	M/F	BW, body composition, cardiometabolic parameters and energy metabolism

*BMI* body mass index, *BW* body weight, *CVD* cardiovascular disease, *F* female, *HDL* high-density lipoprotein cholesterol, *HIIT* High-intensity interval training, *LCD* low-calorie diet, *LDL* low-density lipoprotein cholesterol, *M* male, *NAFLD* non-alcoholic fatty liver disease, *TG* triglycerides, *TRE* time restricted eating

Year of publication ranged from 2016 to 2022, and 78% of the studies had been published in the previous 3 years. The study populations included subjects with normal weight, overweight and obesity. Only one study performed in patients with type 2 diabetes met our inclusion criteria [11], while one study included patients with metabolic syndrome [21] and another non-alcoholic fatty liver disease (NAFLD) patients [37]. The average duration of the TRE protocol was 9.8 weeks. 3 studies were performed in males only, [9, 36, 40] and 3 in females only [20, 23, 35], while the remaining studies were performed in both males and females.

### Qualitative analysis

Sixteen of the twenty studies reported a reduction in fasting glucose after TRE. However, the superiority of TRE vs. control interventions for reducing fasting glucose levels was reported to be significant only by Xie et al. [26], who performed their study in healthy non-obese subjects, and by Che et al. [11], whose subjects had type 2 diabetes. Interestingly, in the studies by Queiroz et al. and Xie et al. in which early TRE (eTRE) and late TRE (lTRE) were analyzed separately, eTRE produced a greater reduction in fasting glucose [25, 26].

HbA1c was evaluated in 10 of the 20 studies. Two studies did not show a greater reduction in HbA1c levels in the TRE group with respect to the control group [4, 22]. In contrast, most of the studies reported a tendency to a decrease in HbA1c in the TRE group, though it did not reach statistical significance [5, 20, 21, 24, 26, 27, 38]. Interestingly, the study by Che et al. performed for 12 weeks in patients with type 2 diabetes with an average HbA1c of 8.5%, demonstrated a much greater reduction in HbA1c in the TRE group than in the control group (p < 0.001; -1.54% and 0.66%, respectively) [11]. With respect to differences between eTRE and lTRE, two studies reported changes in HbA1c and observed similar effects for both eTRE and lTRE interventions [29, 37].

Fifteen studies reported data regarding fasting insulin levels, with heterogeneous results. In 3 studies the control group showed a more marked improvement in insulin levels than the TRE group after the intervention [20, 23, 40]; in fact, Lin et al. reported higher insulin levels in the TRE group post-intervention. The remaining studies found TRE to improve insulin with respect to controls, and in 4 cases the improvement was statistically significant [5, 9, 11, 27]. Of the studies that compared early versus late TRE windows, all showed larger improvements in insulin levels in the former case. [21, 25, 27].

### Meta-analysis

**Fig. 3:**
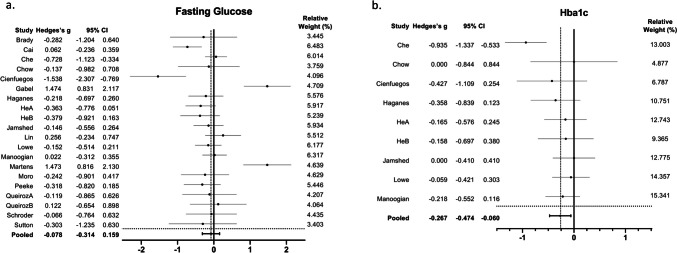
Forest plots of the effect of TRE on **a**. Fasting glucose and **b**. HbA1c, compared to the control group. Suffixes “A” and “B” on author names indicate early TRE and late TRE protocols, respectively, within the same study

**Fig. 4:**
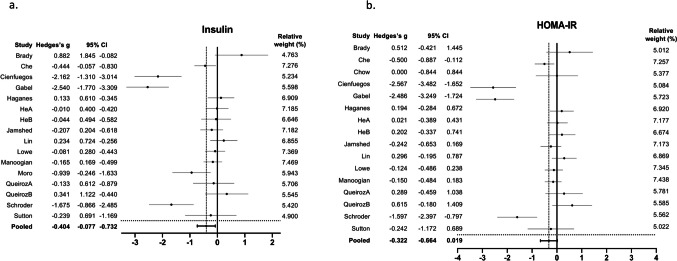
Forest plots of the effect of TRE on **a**. Insulin and **b**. HOMA-IR, compared to the control group. Suffixes “A” and “B” on author names indicate early TRE and late TRE protocols, respectively, within the same study

The results of the meta-analysis are presented in forest plots as Hedges’s g and 95% CI. Hedges's g differences for the individual studies and the pooled overall estimate for fasting glucose and HbA1c are presented in Fig. 3A and B, respectively. Despite TRE having no significant effect on fasting glucose (Hedges’s g = -0.08; 95% CI: -0.31, 0.16; p = 0.56; I^2^ = 24.5%; n = 20), it promoted a significant reduction in HbA1c (Hedges’s g = -0.27; 95% CI: -0.47, -0.06; p = 0.01; I^2^ = 0%; n = 9). TRE also significantly reduced fasting insulin (Hedges’s g = -0.40; 95% CI: -0.73, -0.08; p = 0.01; I^2^ = 41.7%; n = 16) (Fig. 4A), and tended to produce a decrease in HOMA-IR (Hedges’s g = -0.32; 95% CI: -0.66, 0.02; p = 0.06; I^2^ = 43.5%; n = 16) (Fig. 4B).

### Cumulative and subgroup analyses

**Fig. 5:**
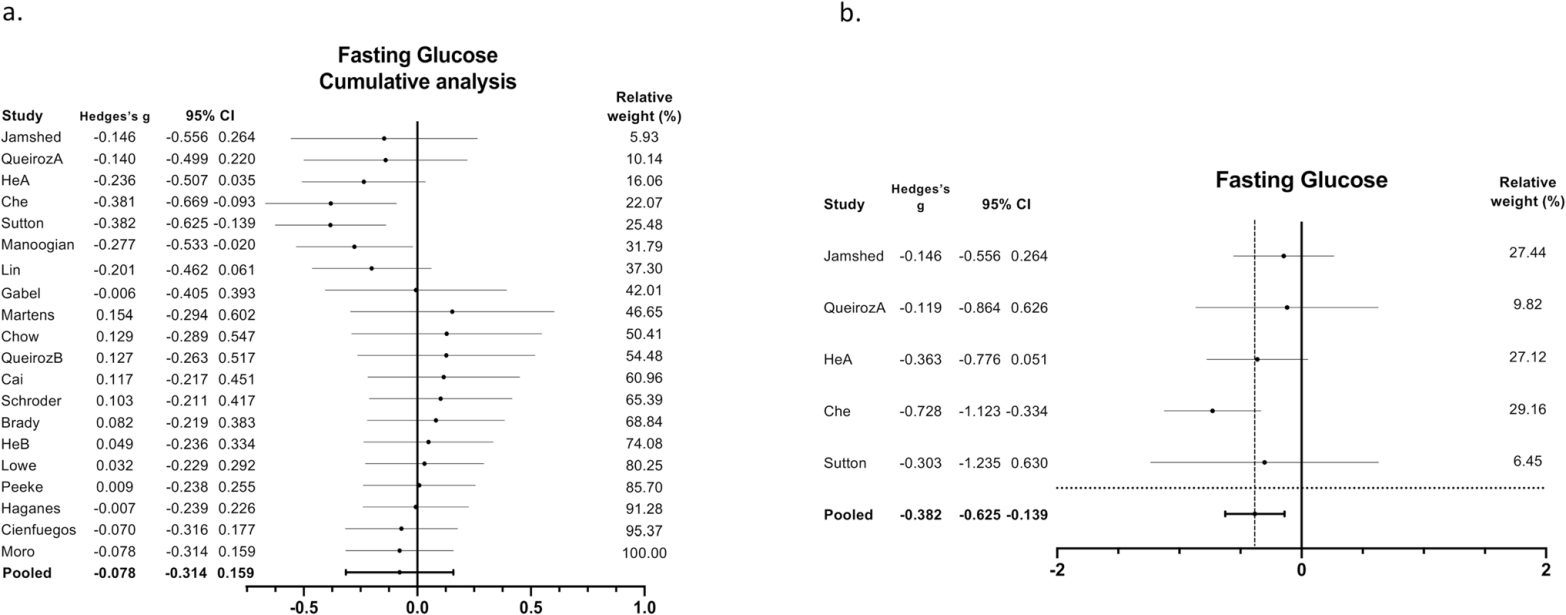
Forest plots summarizing the **a**. Cumulative analysis of fasting glucose according to time of the eating window (earliest to latest) and **b**. Effect of eTRE on fasting glucose. Suffixes “A” and “B” on author names indicate early TRE and late TRE protocols, respectively, within the same study

Our meta-analysis revealed no clear beneficial effect of TRE on fasting glucose levels. However, our systematic review did reveal a potential bias depending on eating window in the TRE group; in other words, the time of the day spanned by the window. Therefore, we decided to assess how early and late TRE windows had influenced the effect sizes of fasting glucose levels reported in the studies. The studies reporting fasting glucose were ordered according to the eating time window (according to the time of breakfast, positioning the studies with the earliest times first) and a cumulative analysis was performed. Interestingly, we observed how effect sizes in terms of glucose levels varied as studies analyzing later TRE windows were included (Fig. 5A). In fact, when we performed a separated analysis for the eTRE studies, fasting glucose was significantly reduced by eTRE compared to controls (Hedges’s g = -0.38; 95% CI: -0.62, -0.14; p < 0.01; I^2^ = 0; n = 5; Fig. 5B).

## Discussion

Our systematic review and meta-analysis reveals that a TRE regimen of a window of between 6 to 10 h during 5 to 14 weeks is sufficient to improve glycemic parameters in subjects with overweight, obesity or type 2 diabetes. In particular, fasting insulin and HbA1c were significantly reduced in the TRE groups versus controls, regardless of calorie restriction or ad libitum regimens. Our analysis also suggests that the timing of food intake is a key factor in TRE dietary regimens, and that eTRE has greater metabolic benefits than lTRE, at least in terms of improving fasting glucose levels in subjects with overweight and obesity. In this sense, a significant reduction in fasting glucose levels was reported only by studies that followed eTRE, in which the regimen consisted of having breakfast before 9am or dinner before 4 pm, results that are in accordance with previous research, in which fasting glucose, insulin and HOMA-IR are reduced specifically by eTRE and not lTRE in healthy subjects [26, 42]. Similarly, in subjects with prediabetes, mean fasting glucose measured by continuous glucose monitoring was decreased by eTRE, and not with lTRE, when compared to baseline [43]. Despite not consisting of a prolonged fasting protocol (13:11 window), the study by Allison et al. demonstrates that eating early in the day versus eating late improves fasting glucose and insulin levels in lean and overweight subjects [44].

Some of the authors reported concerns about adherence to eTRE regimens [45], but recent data by Steger et al. [46] endorses eTRE as a simple dietary intervention that allows subjects to maintain their normal eating habits by concentrating them in a smaller window of time, rather than changing the number, size, or content of meals and snacks.

The beneficial effects of TRE for individuals with prediabetes and diabetes is supported by studies evaluating continuous glucose monitoring, in which an increased time in the normal glucose range upon TRE compared with non-TRE group is witnessed, together with decreased mean 24 h glucose levels [12, 47]. Thus, lowering of HbA1c by TRE is expected, however, the results of our systematic review show that reduction in HbA1c induced by TRE is small, and clinically insignificant. It is important to note that many studies included non-diabetic subjects and that the average HbA1c was 5.5%. In addition, none of the studies lasted more than 14 weeks. These factors may have limited the magnitude of the decrease in HbA1c reported. In fact, the only included study that was performed in patients with type 2 diabetes did show a significant HbA1c decrease of -1.54% in 12 weeks [11].

Another TRE approach that is followed by a large world population and has been extensively studied is Ramadan fasting, in which subjects refrain from eating and drinking from sunrise to sunset for 30 days. Usually they eat two meals a day, one before the sunrise and the other shortly after sunset. Systematic reviews and meta-analyses have shown that Ramadan fasting promoted minimal improvements in fasting glucose in healthy subjects [6], although greater effects were found in subjects with type 2 diabetes [48, 49]. There is a significant but small body weight loss after Ramadan practice [50], though this lost weight is rapidly regained [51]. In addition, other lifestyle changes are modified during the Ramadan period, as for example smoking is forbidden during the daylight hours and sleep duration is reduced due to the overnight eating schedule, since subjects usually fast and work during the daytime [52]. All these factors make difficult to draw conclusions from studies involving Ramadan as a TRE regimen and thus, we have considered excluding them from our systematic review and meta-analysis.

Although some of the studies included in our meta-analysis reported high adherence to the TRE schedule, such as Cai et al., in which > 97% of subjects adhered to the TRE protocol, some of our impressions might have been biased by the inclusion of non-compliant subjects in some of the studies [37]. Brady et al. only included subjects in their final analysis when compliance with the TRE eating window had been greater than 80% [40]. In contrast, the study by Jamshed et al. [22] included a large proportion of non-adherent subjects in both the TRE and control arms, as evidenced by a secondary analysis published by the same group [53]. In this secondary analysis, as expected, the authors reported that the subjects who adhered to the TRE regimen displayed greater improvements in insulin resistance and glucose levels than the general group included in the primary study.

Most of the studies selected for our systematic review allowed ad libitum energy intake. However, some combined TRE with calorie restriction, compared to a control group that followed the same low calorie diet in a 12:12 fasting:feeding time window [22, 23, 25, 41]. Calorie restriction is understood as reducing daily caloric intake by 15–40% without reaching malnutrition, and has classically been associated with improvements in metabolism and increases in longevity [54, 55]. Interestingly, our meta-analysis reveals that, with the exception of one study [23], the effects of TRE exceeded those of calorie restriction alone in terms of reducing fasting glucose [29, 32] and fasting insulin levels [32], especially when a eTRE was adhered to [25].

Among the studies performed in patients with type 2 diabetes, that by Che et al. met our inclusion criteria [11] and was included in the meta-analysis. We found other interesting studies in individuals with type 2 diabetes, such as the one by Parr et al. which we excluded from our systematic review due to the lack of a control group [10]. However, when we compared the post vs. pre changes in fasting glucose and HbA1c in the TRE arm, a significant reduction was evident in both studies, though greater in that by Che et al. (glucose: 26.4 vs 5.4 mg/dl; HbA1c: -1.54% vs 0.2%). The different duration of the TRE interventions (12 weeks in Che et al. vs 4 weeks in Parr et al.) may account for this difference. However, it is important to notice the earlier food intake window followed by subjects in the Che vs Parr study (8am to 6 pm vs 10am to 7 pm), which may have influenced the more pronounced response in glucose and HbA1c.

Ideally, nutrition should be synchronized with clock-regulated metabolic functions. The human body's metabolism is optimized for energy intake early in the day, as glucose tolerance and insulin sensitivity are higher upon waking than in the evening [56], partially because of the suppressive effect of melatonin on insulin secretion [57]. Molecular clocks in metabolic tissues such as adipose tissue or skeletal muscle are regulated by food intake. A clear example is the liver, where the peripheral clock system synchronizes gluconeogenesis and glucose release with the habitual fasting period [58]. Moreover, it should not be forgotten that our human ancestors were not sedentary and did not eat snacks or several meals during the day. In this respect, humans have evolutionary conserved cellular responses to adapt to prolonged fasting periods and physical activity [59]. For all these reasons, the TRE paradigm should be physiologically advantageous by acting on multiple organ systems.

Recent evidence suggests that most of the metabolic benefits of TRE are independent of weight loss [60]. Sutton et al. clearly demonstrated that TRE exerts beneficial effects on insulin sensitivity and improves beta-cell function without altering body weight [9]. In addition, a study by Andriessen et al. [12] reported modest effects of TRE on weight loss, but significant improvements in 24 h mean and fasting glucose, as well as glucose time in range after only 3 weeks of intervention. Given the crossover design of both studies, and despite the small sample sizes, their results are of great value. Mechanisms underlying glucose metabolism improvements by TRE besides body weight loss are not likely related to peripheral and hepatic insulin sensitivity or lipid content, muscle mitochondrial function, or changes in energy metabolism [12]. Instead, TRE has been described to affect the rhythmicity of serum and muscle amino acid and lipid metabolites and to regulate the rhythmicity of genes controlling amino acid transport [61]. Nevertheless, further research is warranted to decipher the molecular mechanisms triggered by TRE and that contribute to the metabolic health improvements.

Our findings highlight that, in the context of TRE, eating early in the day confers greater benefits on some glycemic parameters than delaying eating times. This is in line with a recent review by Lotti et al. [62] in which subjects with evening choronotypes were associated with a worse cardiometabolic risk profile and higher risk of diabetes. Considering that TRE does not necessarily imply a reduction of calorie intake, one can assume that confining all caloric intake to within a defined time window of a few hours has multiple health benefits, particularly if that window is early in the day. Further research is needed in subjects with prediabetes and type 2 diabetes to demonstrate whether greater benefits can be achieved.

### Supplementary Information

Below is the link to the electronic supplementary material.

Supplementary file1 (PDF 130 KB)

Supplementary file2 (XLSX 128 KB)

Supplementary file3 (PDF 162 KB)

## Data Availability

All data analysed during this study are included in this published article and its supplementary information files.

## Abbreviation

BMI: Body mass index
eTRE: Early time-restricted eating
HOMA-IR: Homeostasis model assessment of insulin resistance
IF: Intermittent fasting
lTRE: Late time-restricted eating
NAFLD: Non-alcoholic fatty liver disease
RCT: Randomized controlled trial
SD: Standard deviation
TRE: Time-restricted eating
