# Supplementary material for: Circadian alignment of food intake and glycaemic control by time-restricted eating: A systematic review and meta-analysis
Source: Rev Endocr Metab Disord. 2023 Nov 22;25(2):325–37. doi: 10.1007/s11154-023-09853-x (PMC10943166; doi:10.1007/s11154-023-09853-x)
Supplement: Supplementary file 1 — Supplementary file1 (PDF 130 KB) [file 11154_2023_9853_MOESM1_ESM.pdf]

Reviews in Endocrine and Metabolic Disorders

Circadian alignment of food intake and glycaemic control by time-restricted eating: a systematic review and meta-analysis

Rovira-Llopis S [1,2]*, Luna-Marco C [2], Perea-Galera L [2], Bañuls C [2], Morillas C [2], Victor VM [1,2,3]*.

[1] Department of Physiology, School of Medicine, University of Valencia and Institute of Health Research INCLIVA, Valencia, Spain.

[2] Service of Endocrinology and Nutrition, University Hospital Doctor Peset, Foundation for the Promotion of Health and Biomedical Research in the Valencian Region (FISABIO), Valencia, Spain.

[3] CIBERehd - Department of Pharmacology, University of Valencia, Valencia, Spain; Foundation for the Promotion of Health and Biomedical Research in the Valencian Region (FISABIO), Valencia, Spain.

* Correspondence: susana.rovira@uv.es; victor.victor@uv.es

Tel.: +34 963-188-867 (SRL); +34 961-625-739 (VMV) Fax: +34-961-622-492.

PUMBED SEARCH

("time-restricted eating" OR "time-restricted feeding" OR "prolonged nightly fasting" OR "prolonged overnight fasting") AND ("glycemic control" OR "HbA1c" OR "glucose" OR "diabetes")

Embase

Session Results

....

No. Query

Results   Results Date

#1. ('time-restricted eating' OR 'time-restricted   418 1 Jan 2023

feeding'/exp OR 'time-restricted feeding' OR 'prolonged nightly fasting' OR 'prolonged overnight fasting') AND ('glycemic control'/exp OR 'glycemic control' OR 'hba1c'/exp OR 'hba1c' OR 'glucose'/exp OR 'glucose' OR 'diabetes'/exp OR 'diabetes')

....

Cochrane Library

Search Name:

Date Run: 01/01/2023 23:09:13

Comment:

ID	Search	Hits
#1	"time-restricted eating" OR "time-restricted feeding" OR "prolonged nightly fasting" OR "prolonged overnight fasting"	274
#2	"glycemic control" OR "HbA1c" OR "glucose" OR "diabetes"	137874
#3	{AND #1- #2}	157
