# Supplementary material for: Circadian alignment of food intake and glycaemic control by time-restricted eating: A systematic review and meta-analysis
Source: Rev Endocr Metab Disord. 2023 Nov 22;25(2):325–37. doi: 10.1007/s11154-023-09853-x (PMC10943166; doi:10.1007/s11154-023-09853-x)
Supplement: Supplementary file 3 — Supplementary file3 (PDF 162 KB) [file 11154_2023_9853_MOESM3_ESM.pdf]

Reviews in Endocrine and Metabolic Disorders

Circadian alignment of food intake and glycaemic control by time-restricted eating: a systematic review and meta-analysis

Rovira-Llopis S [1,2]*, Luna-Marco C [2], Perea-Galera L [2], Bañuls C [2], Morillas C [2], Victor VM [1,2,3]*.

[1] Department of Physiology, School of Medicine, University of Valencia and Institute of Health Research INCLIVA, Valencia, Spain.

[2] Service of Endocrinology and Nutrition, University Hospital Doctor Peset, Foundation for the Promotion of Health and Biomedical Research in the Valencian Region (FISABIO), Valencia, Spain.

[3] CIBERehd - Department of Pharmacology, University of Valencia, Valencia, Spain; Foundation for the Promotion of Health and Biomedical Research in the Valencian Region (FISABIO), Valencia, Spain.

* Correspondence: susana.rovira@uv.es; victor.victor@uv.es

Tel.: +34 963-188-867 (SRL); +34 961-625-739 (VMV) Fax: +34-961-622-492.

Supplementary Table 2. Characteristics and main findings of the studies excluded from the meta-analysis due to lack of extractable data.

Author (year)	Study design and groups	Population	Duration (weeks)	TRE hours Fast:Fed	Calorie Restriction	Eating window	Control group	n (control/ TRE)	Age group	Sex	Main findings on outcomes of interest
Xie (2022)	Randomized, C/eTRE/mTRE	Healthy non-obese	5	16:8	No (*ad libitum*)	eTRE: 6am-2pm; mTRE: 11am-8pm	Ad libitum no TRE	28/e28/ m26	Young	M/F	Only eTRE and not mTRE decreased significantly fasting glucose levels and HOMA-IR, compared to the control group. No significant change in HbA1c.
Zhang (2022)	Randomized, C/eTRE/lTRE	Overweight/ obese (BMI ≥ 24 kg/m^2)	8	18:6	No (habitual diet)	eTRE 7am-1pm; lTRE 12pm-6pm	Habitual diet	19/e21/l 20	Young	M/F	eTRE, and not lTRE, reduced significantly fasting insulin, HOMA-IR and mean glucose assessed by continuous glucose monitoring. No significant change in HbA1c.

BMI: body mass index; eTRE: early time restricted eating; F: female; lTRE: late time restricted eating; mTRE: mid-day time restricted eating; M: male; TRE: time restricted eating
